# Mirror visual feedback during unilateral finger movements is related to the desynchronization of cortical electroencephalographic somatomotor alpha rhythms

**DOI:** 10.1111/psyp.14116

**Published:** 2022-06-03

**Authors:** Marco Rizzo, Laura Petrini, Claudio Del Percio, Susanna Lopez, Lars Arendt‐Nielsen, Claudio Babiloni

**Affiliations:** ^1^ Center for Neuroplasticity and Pain (CNAP), SMI^®^ Department of Health Science and Technology Aalborg University Aalborg Denmark; ^2^ Department of Physiology and Pharmacology “V. Erspamer” Sapienza University of Rome Rome Italy; ^3^ Department of Medical Gastroenterology, Mech‐Sense Aalborg University Hospital Aalborg Denmark

**Keywords:** alpha rhythm, EEG, eventrelated desynchronization/synchronization (ERD/ERS), mirror visual feedback (MVF), motor cortex, time‐frequency analysis

## Abstract

Using a mirror adequately oriented, the motion of just one hand induces the illusion of the movement with the other hand. Here, we tested the hypothesis that such a mirror phenomenon may be underpinned by an electroencephalographic (EEG) event‐related desynchronization/synchronization (ERD/ERS) of central alpha rhythms (around 10 Hz) as a neurophysiological measure of the interactions among cerebral cortex, basal ganglia, and thalamus during movement preparation and execution. Eighteen healthy right‐handed male participants performed standard auditory‐triggered unilateral (right) or bilateral finger movements in the *No Mirror (M−)* conditions. In the *Mirror (M+)* condition, the unilateral right finger movements were performed in front of a mirror oriented to induce the illusion of simultaneous left finger movements. EEG activity was recorded from 64 scalp electrodes, and the artifact‐free event‐related EEG epochs were used to compute alpha ERD. In the *M−* conditions, a bilateral prominent central alpha ERD was observed during the bilateral movements, while left central alpha ERD and right alpha ERS were seen during unilateral right movements. In contrast, the *M+* condition showed significant bilateral and widespread alpha ERD during the unilateral right movements. These results suggest that the above illusion of the left movements may be related to alpha ERD measures reflecting excitatory desynchronizing signals in right lateral premotor and primary somatomotor areas possibly in relation to basal ganglia‐thalamic loops.

## Abstract 2

This researche aimed to unveil the cortical areas and neurophysiological mechanisms underlying the Mirror Visual Feedback illusory phenomenon. The analysis of the scalp EEG alpha ERD demonstrated a bilateral activity of the motor and posterior parietal cortex during unilateral mirrored finger movements, likely related to the ganglia‐thalamo‐cortical motor loop typically observed during actual movements. Enhancing our knowledge might be useful to developing therapies for those patients with motor diseases.

## 1 INTRODUCTION

Mirror visual feedback (MVF) illusion techniques, such as mirror box therapy and, in recent years, its implementation using immersive visual reality, were suggested to treat some chronic pain conditions (Murray et al., 2006; Mercier & Sirigu, 2009; Ortiz‐catalan et al., 2016; Thøgersen et al., 2020; for a review, see Lamont et al., 2011). Those conditions include phantom limb pain (PLP, Ramachandran et al., 1995; Ramachandran & Rogers‐Ramachandran, 1996; Chan et al., 2007; Finn et al., 2017), complex regional pain syndrome (McCabe et al., 2003; Vladimir Tichelaar et al., 2007), and poststroke paralysis or stiffness (Bae et al., 2012; Bartur et al., 2018; Dohle et al., 2009).

Ramachandran's mirror box therapy is probably the most well‐known MVF technique (Molla & Boulic, 2013) used. It entails placing a mirror in the midsagittal plane in front of the subject, with the affected limb covered by the box and the non‐affected limb reflected on the mirror in the subject's visual space coincident with the expected position of the affected one. Moving the non‐affected limb in front of the mirror gives the illusion of the other limb movement (Ramachandran & Rodgers‐Ramachandran, 1996). It has been proposed that the illusion of the movement with the affected (immobile) limb is due to an activation of the contralateral sensorimotor cortex even if that limb is still indeed during the illusory movement (Makin & Flor, 2020; Molla & Boulic, 2013).

To understand the underlying cortical mechanisms and the involved brain areas, MVF illusion techniques were applied in several experimental studies in neurologically healthy individuals. Some of these studies used brain stimulation of the primary motor cortex (M1) and neuroimaging techniques to enhance the spatial resolution of the results. However, those results were contrasting. For instance, studies using transcranial magnetic stimulation to probe the excitability of the primary motor cortex during motor tasks performed with the MVF illusion techniques did show enhanced motor evoked responses over the primary motor cortex ipsilateral to the moving limb inducing illusory movements of the other limb (Aziz‐Zadeh et al., 2002; Fukumura et al., 2007; Garry et al., 2005). Similarly, studies using functional magnetic resonance imaging (fMRI) found M1 activity contralateral to the observed hand in the mirror (Numata et al., 2013; Shinoura et al., 2008; Merians et al., 2009). In contrast, other studies using fMRI, transcranial magnetic stimulation, and near‐infrared spectroscopy failed in finding an activation of the M1 contralateral to the reflected hand movement (Fritzsch et al., 2014; Funase et al., 2007; Mehnert et al., 2013), whereas an experiment using magnetoencephalography found an enhanced M1 activity only when the active hand was covered (Hadoush et al., 2013).

Several studies used electroencephalography (EEG) techniques, which probe the movement‐related activity of primary sensorimotor areas with less spatial resolution but fine temporal resolution (Lee et al., 2015). In a series of EEG studies in healthy participants, Touzalin‐Chretien and colleagues used standard MVF illusion techniques and showed that dominant visually triggered lateralized readiness potentials (LRPs, an electrophysiological feature of premotor activation in the M1) were generated in the scalp central region ipsilateral to the hand movement inducing the illusion of the movement in the other (immobile) hand (Touzalin‐Chretien et al., 2009, 2010; Touzalin‐Chretien & Dufour, 2008). In another study, this dominance of the LRPs was not observed by Praamstra et al. (2011) using a similar procedure. However, they confirmed that LRPs reflected some minor activity of the M1 ipsilateral to the hand movement inducing the illusion of the movement in the other (immobile) hand (Praamstra et al., 2011).

EEG techniques can probe another important dimension of the neurophysiological mechanisms underpinning the preparation and execution of human movements, namely the so‐called event‐related desynchronization/synchronization (ERD/ERS) of scalp EEG alpha rhythms at about 8–12 Hz (Pfurtscheller & Lopes Da Silva, 1999). These rhythms are prominent in central and posterior scalp regions as a sign of inhibition of somatomotor and visual cortical regions during a resting state eyes‐closed condition and psychophysical relaxation (Babiloni et al., 2020). During the preparation and execution of movements, alpha rhythms show a significant ERD (reduction in amplitude) in central and parietal regions reflecting the activation of somatomotor cortical areas (Babiloni et al., 1999; Pfurtscheller & Lopes Da Silva, 1999; Pfurtscheller & Neuper, 2006). Thus, the preparation and the execution of a voluntary movement provoke an alpha event‐related desynchronization (ERD) in the brain motor areas, representing a pattern of cortical activity (Babiloni et al., 2010). Notably, such an alpha ERD was shown even during motor imagery and own hand action observation (Duann & Chiou, 2016; Nagai & Tanaka, 2019; Pfurtscheller & Lopes Da Silva, 1999).

Other studies using the MVF extended the focus of the cortical activity analysis on other brain regions. Bartur et al. (2015) found a bi‐hemispheric EEG cortical activity distributed over the fronto‐parietal areas while healthy participants performed wrist extension tasks with the use of the MVF, as compared to the unilateral cortical activity (contralateral to the movement) when the same participants performed the movements without the mirror illusion. Similarly, an fMRI study using a mirror/no‐mirror with hand movements paradigm sin healthy subjects found a strong activity of the parietal‐occipital area in the mirror condition rather than the no‐mirror condition (Matthys et al., 2009). Such an activity spread around the scalp, which involves brain regions ipsilateral to the moving limb, suggests a remarkable role of the fronto‐parietal and parieto‐occipital networks in the elaboration of illusory movements–as shown by many MVF studies (Bartur et al., 2015; Fritzsch et al., 2014; Matthys et al., 2009; Mehnert et al., 2013).

To our knowledge, one EEG study used also MVF illusion techniques and the analysis of anticipatory alpha ERD in healthy individuals (Lee et al., 2015). In that study, the Authors applied a digital MVF technique to produce 2 s‐delays in the illusion of the moving hand in the mirror (indeed still) during a paradigm of self‐paced button press movements with an inter‐movement interval of 15–20 s. Results showed stronger central alpha ERD over bilateral motor cortices in the MVF condition as compared to the baseline without MVF; this effect was delayed for about 2 s when the illusory movement was delayed by the system (Lee et al., 2015). However, the use of self‐paced unilateral movements and only three EEG recording channels (e.g., C3, Cz, and C4) represented a significant limitation in the analysis of the MVF effects on central alpha ERD. Indeed, the placement of only three electrodes on the scalp central areas did not allow the appropriate localization of maximum alpha ERD during the MFV illusion of movements. To address these issues, we designed the present study.

This study aimed to use a standard MVF technique to test the hypothesis that the mentioned mirror phenomenon may be underpinned by a prominent central alpha ERD. As a methodological step forward, the use of auditory‐triggered finger movements allowed the mitigation of the bilateral central alpha ERD preceding self‐paced movements. Furthermore, a montage with 64 scalp electrodes allowed a fine topographical mapping of alpha ERD, extending the analysis to wider frontal, and centro‐parietal cortical areas. Finally, the inclusion of both control unilateral and bilateral finger movements allowed the localization of maximum alpha ERD in the scalp in both hemispheres in relation to our specific experimental conditions.

The hypothesis was that the MVF illusion of left finger movements during true right finger movements may be related to a significant alpha ERD in the right central area. Secondarily, through the analysis of the alpha ERD/ERS within the frontal and parietal areas, we attempted to provide an overview of antero‐posterior visuo‐motor networks involved in the elaboration of illusory movements.

## 2 METHOD

### 2.1 Participants

All experiments were conducted at the Aalborg University (Denmark). Eighteen right‐handed male healthy volunteers (mean age = 28.7, *SD* = ±4.9) participated in the study. Exclusion criteria included chronic pain, nerve pain, neurological and psychiatric diseases, and chronic medical treatments. Inclusion criteria included right‐handedness as revealed by The Edinburgh Handedness Inventory (EHI, Oldfield, 1971). Only right‐handed subjects were recruited, as previous studies showed that handedness might affect the illusion experience (Niebauer et al., 2002). All participants signed an informed consent form according to the Declaration of Helsinki (they were allowed to interrupt the trial at any time). The study was approved by the Scientific Ethical Committee of Region Nordjylland (N‐20190008).

### 2.2 Experimental procedure

The participants were comfortably seated on a chair with both arms placed symmetrically ahead on a desk. Auditory tones of 70 dB, 1000 Hz, and 50 ms of duration (Kida et al., 2006) were delivered to trigger the movements. The interval between the auditory stimuli was fixed at 10 s, a sufficient period to reset the synchronization of the alpha rhythms (Babiloni et al., 2008). Three conditions of 80 trials each were randomized across the participants.

**FIGURE 1:**
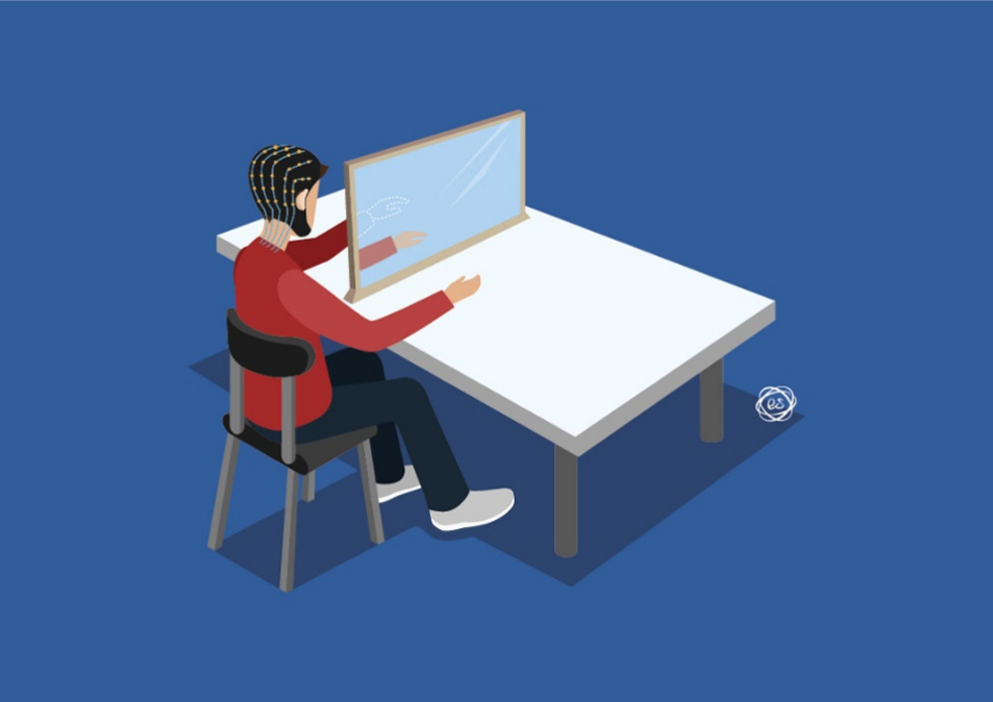
Illustration of the mirror visual illusion (MVF) procedure in the experimental setting during the unilateral mirror (*M+*) condition. The left arm is placed behind the mirror (dotted line), out of the subject's sight. The right arm is reflected in the mirror, giving the illusion of ownership of the mirrored arm. In the control conditions–unilateral no mirror (*M−*) and bilateral no Mirror (*M−*)–the mirror was removed, and the left arm was exposed to the subject's sight. EEG was recorded during all conditions.

In the experimental condition, *Unilateral Mirror (Uni M+)*, a mirror was placed on the desk, in the midsagittal plane in front of the subject with the reflecting face on the right side (Figure 1). The participants were instructed to perform a movement in response to an auditory signal. The movement consisted of a double extension of the right index finger (between 30° and 45°) with a slow release toward the bottom (approx. 1 s). In this condition, the movements were performed in front of the mirror to induce the illusion of the left finger movement. The left hand was kept behind the mirror in a symmetrical position with respect to the right hand. Subjects were instructed to keep their left hand as still and relaxed as possible. Noteworthy, all participants received a brief successful training to perform a stable motor performance of the fingers' movements and not to perform left hand movements during the execution of right finger movements in the *Unilateral* conditions. Furthermore, the experimenters controlled the eventual occurrence of muscles twitch or small involuntary movements of the left hand to alert the participants. In the control conditions, the mirror was removed from the experimental setting (no illusory movement), and the left hand was visible to the participants. They were asked to perform auditory‐triggered hand movements. In one control condition, *Unilateral No Mirror (Uni M−)*, the unilateral right movements were required, while they kept the left hand as still and relaxed as possible. In this condition, all participants also received a brief successful training not to perform left hand movements during the execution of right finger movements. In the other control condition, *Bilateral No Mirror (Bil M−)*, synchronous bilateral movements were required.

### 2.3 EEG recording and analysis

The EEG recordings were performed in the late morning. The EEG activity was collected from 64 scalp electrodes by an active 64‐channel system (g.HIamp amplifier, g.tec medical engineering GmbH, Austria). The scalp electrodes were mounted on a standard elastic cap according to the 10–10 international system. Reference and ground electrodes were positioned on the ear lobes interconnected and on the forehead, respectively. The scalp electrodes impedance was kept under 5 kΩ. The sampling rate was 1200 Hz with anti‐aliasing filters. Eye movements and blinking activity were detected by Fp1 and Fp2 channels (Zeng et al., 2013).

The offline EEG data analysis was performed by the EEGLAB 2021.1 (Delorme & Makeig, 2004) freeware toolbox for MatLab R2018b (MathWorks, Inc., Natick, MA). All EEG data were visually inspected, filtered with a zero‐phase basic FIR filter (0.3–40 Hz), resampled (to 256 Hz), re‐referenced, and epoched in 80 9‐s windows. In all EEG epochs, artifacts (e.g., eyes movements, blinking, head or facial movements, 50 Hz noise, etc.) were recognized and removed using the Infomax independent component analysis (ICA) algorithm of Bell and Sejnowski (1995). On average, 10.8% of artifacted epochs, and 10.5% of artifacted components were removed from the EEG datasets.

To quantify alpha ERD/ERS from artifact‐free EEG epochs, the global EEG power spectrum was computed using an FFT‐based method. The results were used to select the individual alpha frequency (IAF) peak from EEG power spectra, defined as the frequency showing the highest power peak within the alpha range (about 8–12 Hz) (Klimesch, 1999). Finally, the EEG data were individually filtered at the alpha frequency band depending on the IAF, namely from IAF‐2 Hz to IAF + 2 Hz (mean IAF peak = 10.2; *SD* = ±1.0).

### 2.4 ERD/ERS calculation

A standard quantification of the alpha ERD/ERS was performed for the anticipation and the execution of the movements (Babiloni et al., 2010; Pfurtscheller et al., 1997; Pfurtscheller & Aranibar, 1979; Pfurtscheller & Lopes Da Silva, 1999; Pfurtscheller & Neuper, 1994). The alpha ERD/ERS was calculated by the following formula: ERD/ERS% = (E‐R)/R*100, where E represents the power density at the “event” period (1 s) and R is the power density at the baseline period (1 s). The “rest” period was defined as the period from 5 s to 4 s before the auditory cues. The anticipatory “event” period was defined as the period of 1 s before the auditory cues, whereas the executory “event” period was defined as the period of 1 s from 250 to 1250 ms after the auditory cues. In the execution stage, the alpha ERD peak within the interval 250–1250 ms was considered in the analysis. The first 250 ms after the cues were intentionally removed to exclude the auditory‐evoked potentials (N1‐P2 complex) from the ERD/ERS analysis. The resulting negative percentage values represented the alpha ERD, as a reflection of cortical activity (Pfurtscheller et al., 1997; Pfurtscheller & Lopes Da Silva, 1999). Contrariwise, the positive percentage values represented the alpha ERS. For each hemisphere, clusters of electrodes were considered at frontal (F3 and FC3; F4 and FC4) and centro‐parietal (C3, CP3, and P3; C4, CP4, and P4) levels.

### 2.5 Statistical analysis

For the statistical analysis, repeated measures ANOVAs were performed for the anticipatory and the execution stages of the movement. The fixed factors considered in the two ANOVAs (3 × 2 × 2) were *Condition* (*Uni M*−, *Bil M*−, and *Uni M+*), *Region of Interest*–or *ROI*–(*Frontal* and *Central*), and *Hemisphere* (*Left* and *Right*).

Sphericity assumption was checked with Mauchly's test and Greenhouse–Geisser degrees of freedom correction was considered when necessary. Pairwise post hoc planned comparisons were Bonferroni corrected. The working hypothesis predicted a three‐way effect of the ANOVA and post hoc tests showing a significant difference in alpha ERD between *ROI* and *Hemisphere* levels in the *Uni M−* and no differences in the *Bil M−* and *Uni M+* (*p* < 0.05, corrected). We planned to split the 3 × 2 × 2 ANOVA into two 3 × 2 ANOVAs by the factor *ROI* if the latter showed any significant interaction with the *Condition* and *Hemisphere* factors.

### 2.6 Control analysis

Bayes Factor (BF) paired samples *t*‐tests were applied to test the hypothesis that–despite the unilateral right finger movement–no statistically significant differences were observable between the left and right central alpha ERD amplitude means (i.e., cortical activity) in the experimental condition (*Uni M+*), during the anticipation stage. The default Cauchy prior (0, r = 1/√2) was used for the individual comparisons, and the outcomes were ranked accordingly to Lee and Wagenmakers' classification (2013). BF analysis was performed using the statistical freeware JASP (v. 0.16.1.0).

Moreover, through the Grubbs' test (arbitrary threshold of *p* < 0.01) we controlled whether the effects of the statistical analysis might be due to the presence of outliers in the anticipatory alpha ERD/ERS and alpha ERD peak during the movement execution values.

Finally, for better control of the eventual occurrence of muscle twitches or small involuntary movements of the left hand, ongoing surface electromyographic (EMG) activity was recorded from the left and right hands during the finger movements (for further details, see Supplementary Materials). A two‐way ANOVA for repeated measures was performed for the EMG data, considering *Condition* (*Unilateral M−, Bilateral M−,* and *Unilateral M+*) and *Hand* (*Left* and *Right*) as factors (*p* < 0.05). The working hypothesis predicted a two‐way effect of the ANOVA and post hoc tests showing a significant difference in the EMG measures in the left finger between *Bilateral M−* and *Unilateral M+* and *Unilateral M−* (p < 0.05, corrected) and no differences in the left finger between *Bilateral M−* and *Unilateral M+* (p > 0.05, corrected).

## 3 RESULTS

### 3.1 Alpha ERD/ERS topography

**FIGURE 2:**
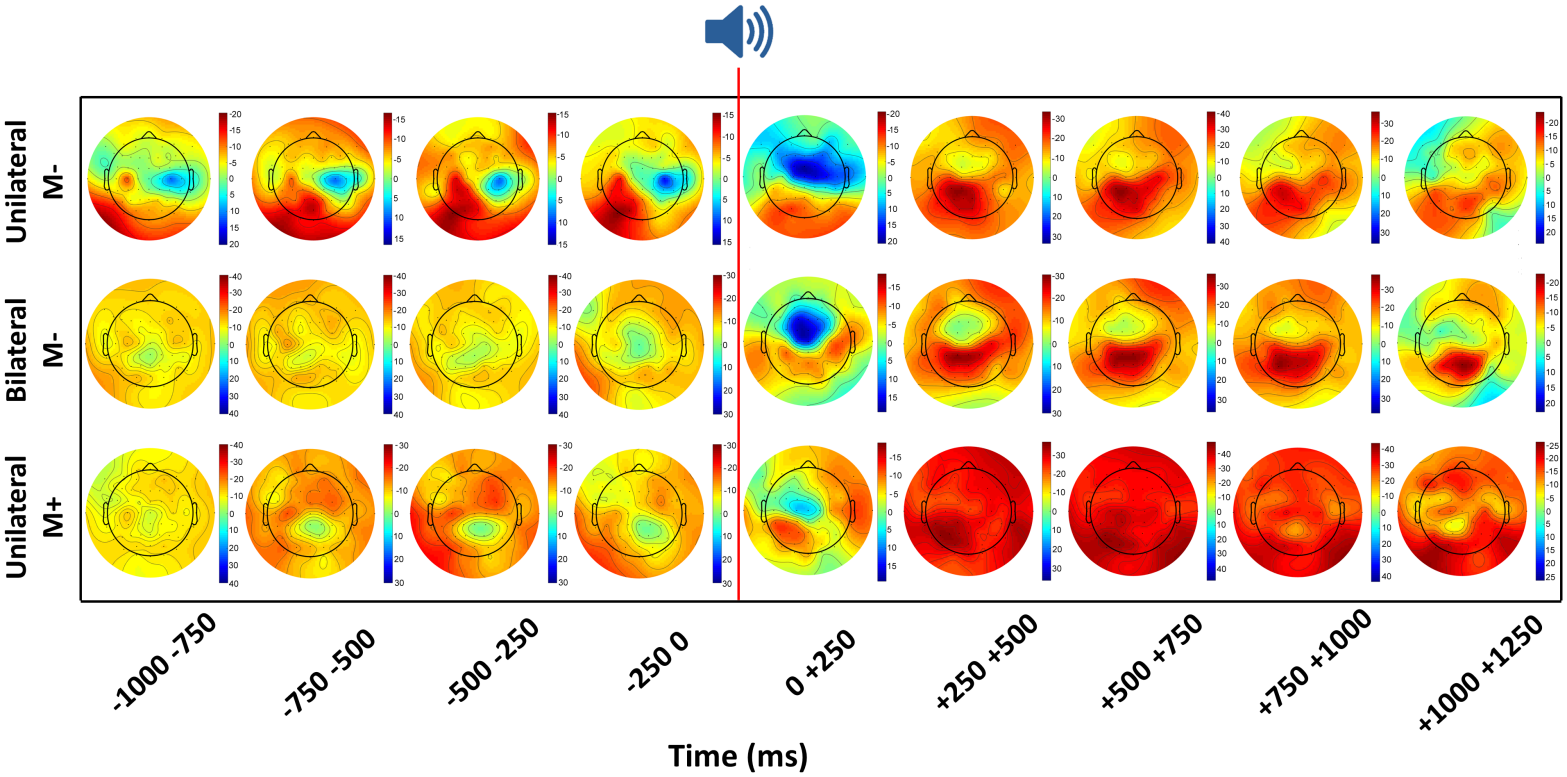
Across subjects mean 2‐D maps of anticipatory alpha ERD/ERS distribution over the scalp for each condition (unilateral *M−*, bilateral *M−*, and unilateral *M+*). The graph starts from 1000 ms before the auditory cue (red line, time = 0) and ends at 1250 ms after the cue. The color legends indicate the maximal percent values of ERD (dark red) and ERS (dark blue) of each map.

The topographic maps of the alpha ERD/ERS in the *Unilateral M−, Bilateral M−*, and *Unilateral M+* conditions (Figure 2) represented the time interval from 1000 ms before the auditory cue to 1250 ms after the cue. In the *Uni M−* condition, the right finger movements were associated with an anticipatory alpha ERD prominent in the contralateral scalp frontal, central, and central‐parietal areas, while a clear ipsilateral central anticipatory alpha ERS was observed. In the same condition, the alpha ERD was bilaterally widespread during the movement execution over the central and parietal areas. In the *Bil M−* condition, the bilateral finger movements were related to an anticipatory alpha ERD prominent in the scalp frontal, central, and parietal areas. The same tendency was observed during the movement execution. Similarly, the right finger movements of the *Uni M+* condition showed an alpha ERD prominent at the same bilateral scalp frontal, central, and central‐parietal areas, at both anticipatory and executive stages of the movements. Table 1 reports the mean percentage values of the alpha ERD/ERS during the movement preparation and execution for each condition at the selected regions of interest (frontal left and right, and central left and right).

**Table 1:** ERD/ERS percentage values (mean ± SE) at the alpha band for each condition (unilateral *M−*, bilateral *M−*, and unilateral *M+*) and regions of interest (frontal left and right, and central left and right)

	*Anticipation*			
	*Regions of Interest*			
Conditions	Frontal Left	Frontal Right	Central Left	Central Right
Uni *M*−	−6.15 ± 4.16	−2.29 ± 2.97	−9.88 ± 3.66	6.77 ± 4.89
Bil *M*−	−10.61 ± 4.01	−12.34 ± 3.35	−9.44 ± 3.34	−9.10 ± 3.47
Uni *M*+	−10.15 ± 3.56	−15.32 ± 2.86	−12.61 ± 3.23	−8.09 ± 2.34

	*Execution*			
	*Regions of Interest*			
Conditions	Frontal Left	Frontal Right	Central Left	Central Right
Uni *M*−	−10.96 ± 8.31	−17.60 ± 7.33	−32.28 ± 6.25	−27.50 ± 6.41
Bil *M*−	−11.53 ± 9.67	−18.38 ± 8.06	−30.49 ± 6.97	−27.10 ± 8.03
Uni *M*+	−32.55 ± 5.04	−34.59 ± 4.53	−40.97 ± 4.91	−36.61 ± 5.23

Note: The table includes the anticipation (top) and execution (bottom) phases of the movement.

### 3.2 Statistical analysis

#### 3.2.1 Anticipation

**FIGURE 3:**
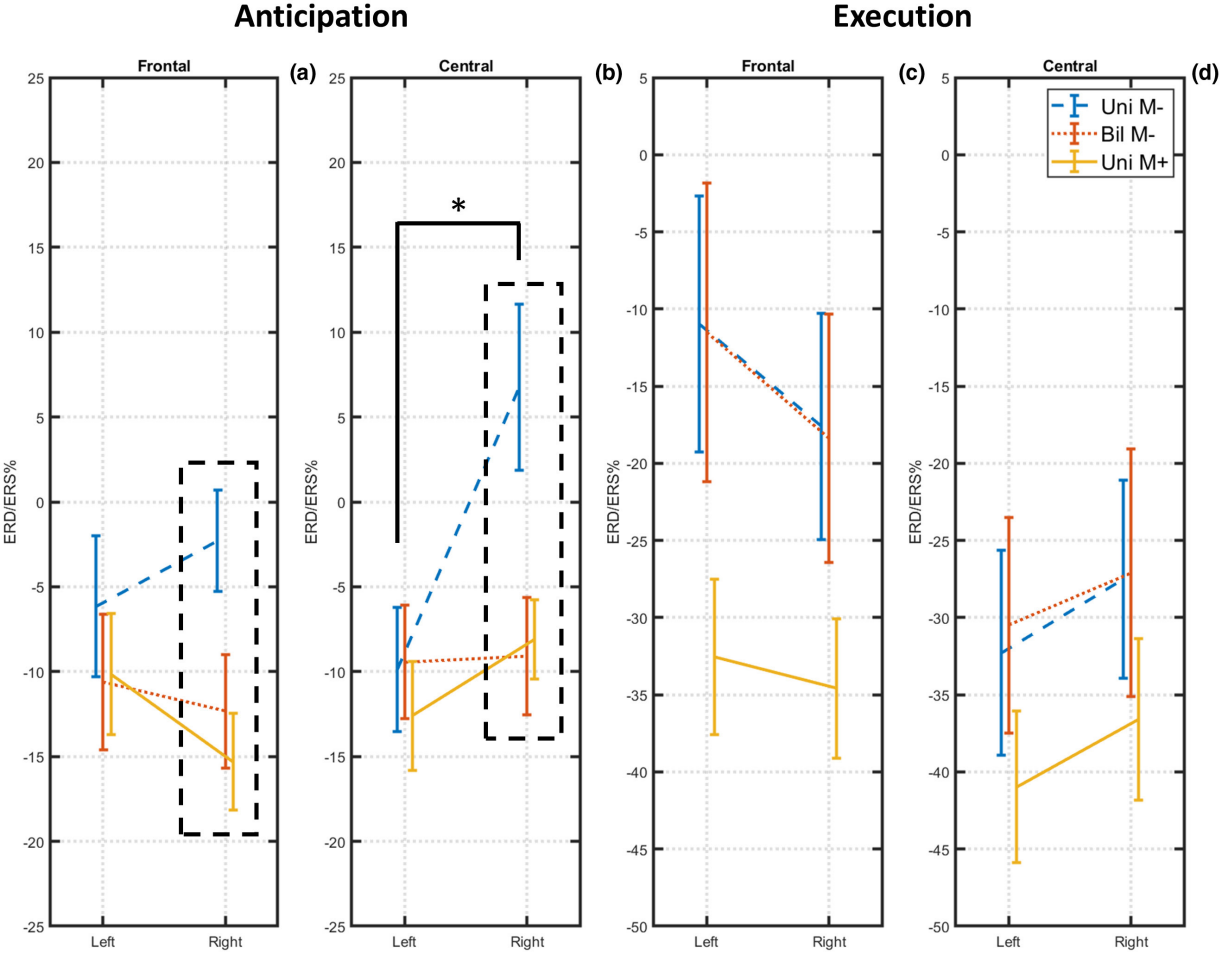
The graph shows the results of the ANOVAs performed for the alpha ERD/ERS during movement preparation and execution (main factors: Condition, ROI, hemisphere). On the Y axis, the mean percent value (± SE) for the Uni *M−* (blue), Bil *M−* (red), and Uni *M+* (yellow) conditions are represented. On the *X* axis, the left and the right hemispheres are represented in each panel. For the anticipatory phase (panels a and b), the alpha ERD over the right hemisphere in the Uni *M−* condition is statistically different from the one in the Bil *M−* and Uni *M+* conditions (p < 0.01) in both the frontal and central ROIs, as showed by Bonferroni post hoc tests. Moreover, in the central ROI, the alpha ERD in the Uni *M−* condition showed significant differences between the left and right hemispheres (*p* = 0.001). For the execution phase (panels c and d), results showed a general stronger alpha ERD in the central ROI as compared to the frontal (*p* = 0.001).

The ANOVA representing the anticipatory interval time (Figure 3a,b) showed a significant result for the main factors *Condition* (F[2,34] = 3343; *p* = 0.047; *η* ^2^ = 0.165) and *Hemisphere* (F[1,17] = 6967; *p* = 0.017; *η* ^2^ = 0.290), and no significant effect for the main factor *ROI* (F[1,17] = 1260; *p* = 0.277). Although the alpha ERD in the *Uni M−* condition appears lower as compared to the other *Bil M−* and *Uni M+* conditions, Bonferroni post hoc tests did not find any statistically significant difference (*p* > 0.05). On the other hand, the alpha ERD is significantly stronger over the left hemisphere when compared to the right (*p* = 0.017), due to the alpha ERS detected over the right hemisphere during the *Uni M−* condition. Moreover, the alpha ERD/ERS pattern remains stable in both the frontal and central ROIs.

The two‐factors interactions found significant results for Cond*Hem (F[2,34] = 10,334; *p* = 0.000; *η* ^2^ = 0.378) and ROI*Hem (F[1,17] = 4930; *p* = 0.040; *η* ^2^ = 0.225), but not for Cond*ROI (*p* = 0.992). Bonferroni post hoc tests showed that the amplitude of the alpha ERD in the right hemisphere (i.e., ipsilateral to the right finger movement) in the *Unilateral M−* condition was significantly lower than that computed in the *Bilateral M−* (*p* = 0.000) and *Unilateral M+* (*p* = 0.000) conditions. In contrast, the anticipatory alpha ERD did not differ between the *Bil M−* and *Uni M+* conditions (*p* = 1.000). Of interest, post hoc tests did not find any statistically significant difference in the alpha ERD amplitude between the left and the right hemisphere neither in the *Bil M−* nor the *Uni M+* conditions (*p* = 1.000). This was true for both the frontal and central ROIs. Despite Bonferroni post hoc tests did not find any statistically significant comparisons for the ROI*Hem interaction, data showed a tendency of a lower alpha ERD over the central ROI of the right hemisphere. Finally, no significant effects have been found from the Cond*ROI*Hem interaction (*p* = 0.253).

The 3x2 ANOVA for the frontal ROI (Figure 3a) did not find significant effects on the main factors *Condition* (*p* = 0.105) and *Hemisphere* (*p* = 0.422), but it did find a significant *Interaction* between the factors (F[2,34] = 5112; *p* = 0.011; *η* ^2^ = 0.231). Bonferroni post hoc tests showed less alpha ERD in the *Uni M−* condition over the frontal right region as compared to the *Bil M−* and *Uni M+* conditions (*p* = 0.000). No differences were found on the left frontal hemisphere among the three conditions. The 3x2 ANOVA for the central ROI (Figure 3b) found a significant effect for the factor *Hemisphere* (F[1,17] = 6219; *p* = 0.23; *η* ^2^ = 0,267), but not for the factor *Condition* (*p* = 0.067). The *Interaction* between the factors was also statistically significant (F[2,34] = 5313; *p* = 0.010; *η* ^2^ = 0,238). Bonferroni post hoc tests showed as the alpha ERD over the central right region is statistically lower in the *Uni M−* condition as compared to the *Bil M−* (*p* = 0.002) and *Uni M+* (*p* = 0.006) conditions. Unlike the frontal, in the central region the alpha ERD amplitude in the *Uni M−* condition was significantly stronger over the left than the right hemisphere (*p* = 0.001).

#### 3.2.2 Execution

The 3x2x2 ANOVA representing the alpha ERD during the execution of the movement (Figure 3c,d) found a statistically significant effect for the factor *ROI* (F[1,17] = 15,664; *p* = 0.001; *η* ^2^ = 0,480), showing a greater alpha ERD amplitude in the central region as compared to the frontal one. This might be explained by a greater engagement of the centro‐parietal brain regions during the motor task. No effects have been found for the factors *Condition* (*p* = 0.070) and *Hemisphere* (*p* = 0.767).

**FIGURE 4:**
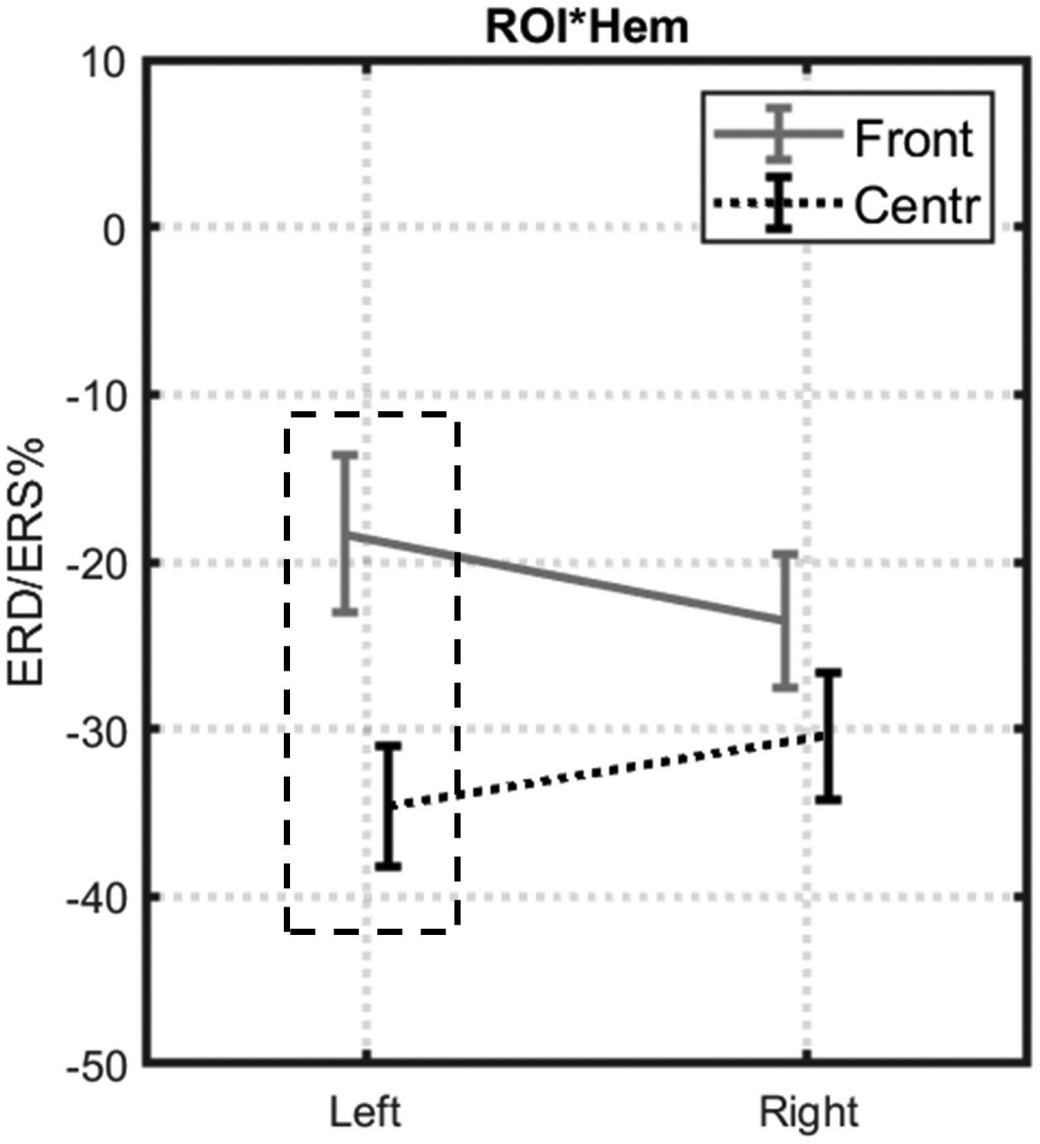
The figure shows the ROI*hemisphere interaction in the execution phase of the movement. On the *Y* axis, the alpha ERD/ERS% (mean ± SE) is represented for the frontal (gray) and central (black) ROIs. The *X* axis indicates the hemisphere (left and right). Bonferroni post hoc tests showed a significantly stronger alpha ERD over the left hemisphere for the Central ROI as compared to the frontal (*p* = 0.000).

The two‐factors interactions resulted significant only for ROI*Hem (F[1,17] = 7060; *p* = 0.017; *η* ^2^ = 0,293). Bonferroni post hoc tests indicated a stronger alpha ERD in the central ROI as compared to the frontal one (*p* = 0.000), only over the left hemisphere (Figure 4). A possible explanation might be represented by a great engagement of the centro‐parietal region during the motor task. However, this difference was not observed in the right hemisphere as only in one condition (*Bil M−*) the alpha ERD was induced by a contralateral actual movement. The Cond*ROI*Hem interaction did not find significant results (*p* = 0.637).

When splitting the ANOVAs by the ROIs, the resultant 3 × 2 ANOVA for the frontal region (Figure 3c) found a significant effect of the factor *Condition* (F[2,34] = 3625; *p* = 0.037; *η* ^2^ = 0,176), indicating a strong alpha ERD in the Uni *M+* condition. However, Bonferroni post hoc tests did not confirm the results. The factor *Hemisphere* (*p* = 0.070) and the Interaction Cond*Hem (*p* = 0.300) were not significant. The 3 × 2 ANOVA for the central region (Figure 3d) found no significant effects for the main factors *Condition* (*p* = 0.211) and *Hemisphere* (*p* = 0.067), nor for the *Interaction* (*p* = 0.928).

### 3.3 Control analysis

Bayesian *t*‐tests confirmed that the alpha ERD amplitude means are not significantly different between the central left and right hemispheres in the *Uni M+* condition for movement preparation. Specifically, the BF_01_ presented a value of 2.236 (Table 2), indicating anecdotal support in favor of the null hypothesis over the alternative hypothesis (i.e., the means are not statistically different).

**Table 2:** Bayesian *t*‐test comparisons from alpha ERD/ERS% (mean ± SE) recorded during the Uni *M+* condition at the central electrodes C3 and C4. The table includes the movement preparation stage. BF = Bayesian factor

	Uni *M*+		
	Central left	Central right	BF_01_
Anticipation	−12.61 ± 3.23	−8.09 ± 2.34	2.236

**FIGURE 5:**
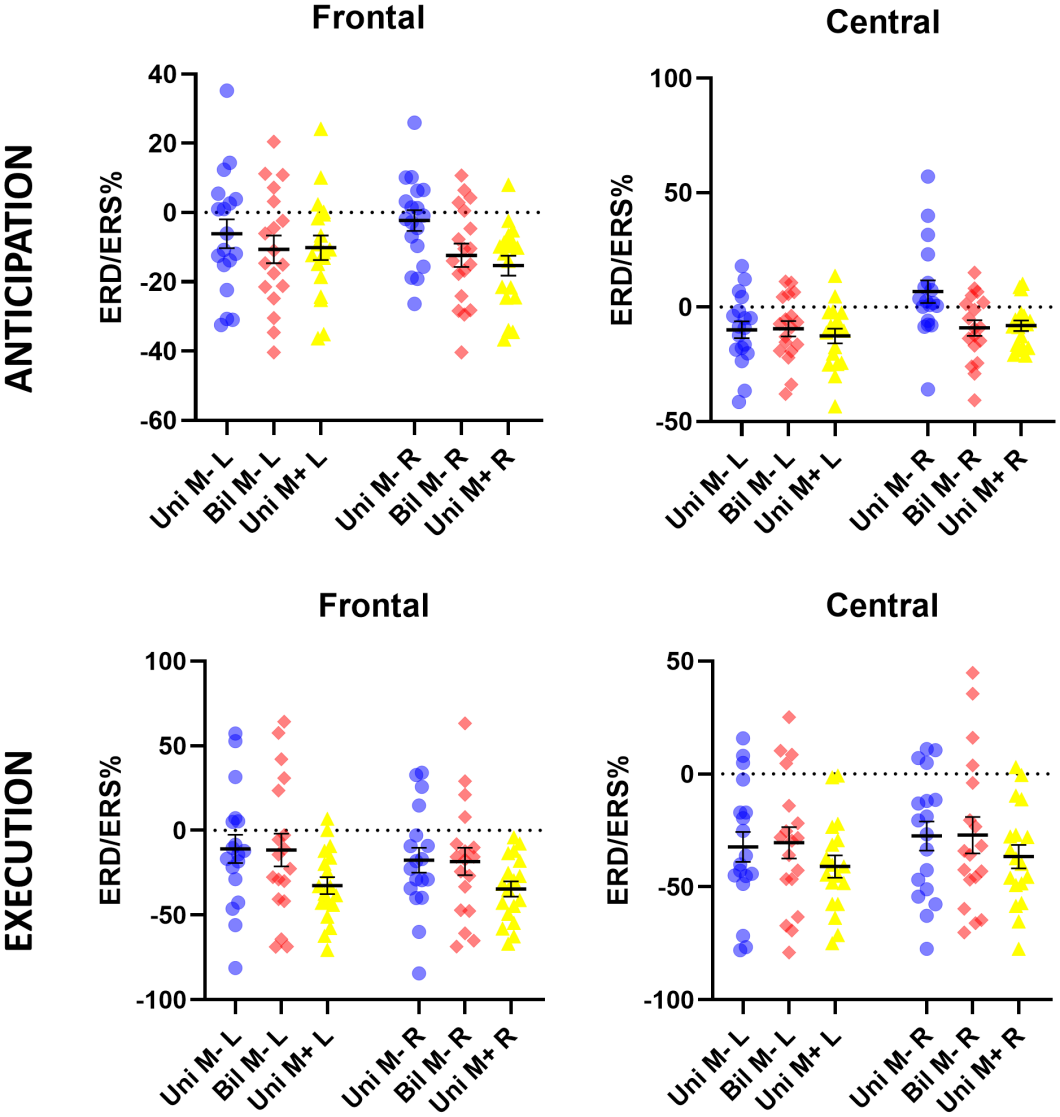
Individual values of the anticipatory alpha ERD/ERS (top panels) and the alpha ERD peak (bottom panels) during the movement for the conditions unilateral *M−* (blue), bilateral *M−* (red), and unilateral *M+* (yellow) and the regions of interest frontal and central. Grubbs' test did not find any outlier with a significance threshold of *p* = 0.01. The black lines represent the mean (± SE) value.

Grubbs' test has not found any outlier value for the anticipatory alpha ERD/ERS and the alpha peak values (Figure 5). Therefore, we assume that the above‐presented results were not affected by outliers on the alpha ERD/ERS percent values.

As mentioned above, the ongoing EMG activity was recorded from the two hands during the EEG experiments carried out on nine participants to control the possible involuntary co‐activation of operating muscles of the left hand during the unilateral auditory‐triggered right finger movements. The results showed as the EMG magnitude recorded from the right hand did not differ between the three conditions. Furthermore, the activities of the right and left hands were similar when recorded in the bilateral (Bil *M−*) condition. Conversely, during the unilateral conditions (i.e., Uni *M−* and Uni *M+*) the EMG activity was negligible over the left hand. Detailed analysis is shown in Supporting Information S1.

## 4 DISCUSSION

Using a mirror adequately oriented, 18 healthy volunteers experienced the MVF illusion of the left finger movement during auditory‐triggered true right finger movements. This study supports the hypothesis that the MVF illusion may be related to an alpha ERD in the right central scalp areas reflecting the underlying excitatory thalamus‐cortical activation of right cortical somatomotor areas, as well as a fronto‐parietal and parieto‐occipital alpha ERD reflecting the involvement of subordinated networks responsible of visuo‐motor elaboration.

The present study succeeded in finding a bi‐hemispheric central alpha ERD during unilateral finger movements, suggesting that the MVF illusion may involve the same cortical mechanisms of a true movement at both preparation and execution stages. This conclusion is corroborated by a more conservative Bayesian control *t*‐test, showing no differences in the alpha ERD amplitude between the left and right centro‐parietal regions during movement preparation. The right motor cortex activation during movement execution in the *Unilateral M−* condition may result contrasting with the initial hypothesis. Nonetheless, our findings are in line with other MVF studies showing a hemispheric asymmetry in the motor alpha ERD/ERS distribution in the control condition (i.e., without the mirror) only during the preparation of the movement rather than the movement execution (Bartur et al., 2015; Li et al., 2018). Another explanation of this spread centro‐parietal alpha ERD might be given by a tactile sensation of the finger on the surface of the table (Spaccasassi et al., 2021). However, previous studies showing a central alpha suppression in response to tactile sensation used high‐frequency vibrotactile stimuli (Kim et al., 2020; Shen et al., 2017; Van Ede et al., 2011). In addition, it should be noted that the bi‐hemispheric alpha ERD observed during the movement execution in the control conditions (*M−*) originates mainly from the central area, whereas it is widespread over the scalp in the experimental condition (*M+*), indicating a great general activation when the mirror is introduced in the experimental paradigm.

### 4.1 The neurophysiological mechanism

At this early stage of the research, it can be assumed that the present results reflect a neurophysiological oscillatory mechanism underpinning visuo‐motor and somatomotor information processing occurring in lateral premotor and primary somatomotor hand areas before and after the auditory stimuli triggering the finger movements, as indicated by a frontal and centro‐parietal alpha ERD.

Before the auditory cue triggering stimuli, pyramidal neurons in lateral premotor and primary somatomotor cortices may generate ample alpha rhythms recordable from electrodes placed on scalp central areas of both hemispheres. Those alpha rhythms, as a component of the so‐called mu rhythms, might be due to synchronizing oscillatory signals (8–12 Hz) conveyed within a feedback loop spanning cortical pyramidal, basal ganglia, and thalamic neurons (Klimesch, 2012; Lopes da Silva, 1991, 2013; Lörincz et al., 2008; Pfurtscheller & Lopes Da Silva, 1999). This state of the neurophysiological mechanism may inhibit visuospatial and somatomotor information flows toward and from those cortical areas (Del Percio et al., 2010; Klimesch, 2012; Lörincz et al., 2008; Pfurtscheller & Lopes Da Silva, 1999).

After the cue triggering the movements, the frontal and the centro‐parietal networks play an important role in the transmission of the visuo‐motor information, as revealed by a widespread alpha ERD over the scalp. In particular, cognitive integrative mechanisms appear linked to the conscious perception of the mirror illusion, as indicated by a stronger frontal alpha ERD during the execution of the movements in the experimental condition. The frontal and parietal areas together are related to the elaboration processing of cognitive information associated with contralateral movement control and perception (Arya, 2016; Serrien et al., 2004). Moreover, the strong alpha ERD observed in the centro‐parietal areas during the movement execution in the *Mirror* condition may reflect the activity of the posterior parietal cortex (PPC). The PPC is linked to the premotor cortex and the Supplementary motor area (SMA), which are responsible for movement control and coordination (Arya, 2016). Results also showed a great involvement of the occipital area during the *Mirror* condition, which is linked to visuo‐motor functions (for a detailed analysis of the occipital electrodes, see Supporting Information Figure S2). In particular, the superior occipital gyrus translates visual information into motor commands through a parieto‐occipital network (Lamont et al., 2011). The parieto‐occipital network–including the superior occipital gyrus–transmits visuo‐motor information to the primary and Supplementary motor areas through the PPC (Arya, 2016).

In the present *Unilateral M+* condition, the true unilateral auditory‐triggered right finger movements and the MVF‐induced illusion of the left finger movements may be related to the desynchronization of pyramidal neurons in bilateral premotor and primary somatomotor areas as well as frontal and centro‐parietal networks, generating the bilateral alpha ERD observed on the scalp. As a consequence, preparatory and executive information processing underlying movement illusion would occur within cortical‐basal ganglia‐thalamo‐cortical loops responsible for the left finger movements (Babiloni et al., 2008; Pfurtscheller & Lopes Da Silva, 1999; Ribary et al., 2017). The anticipatory alpha ERD prominent in the central area contralateral to the illusory movement and the widespread alpha ERD observed during the movement execution in the *Mirror* condition corroborate the hypothesis of a bi‐hemispheric cortical activity due to the MVF illusion.

### 4.2 Clinical relevance

From a clinical perspective, these findings may help to develop mirror‐ and EEG‐based motor therapies. Indeed, previous studies showed anticipatory central alpha ERD associated with movement observation and motor imagery (Duann & Chiou, 2016; Gonzalez‐Rosa et al., 2015; Wriessnegger et al., 2018). Other studies clarified the significant role of the neurophysiological mechanisms generating central alpha ERD induced by motor imagery for the control of prosthesis or mechanical devices by brain computer interface (BCI) (Tang et al., 2016). In particular, identifying the individual central alpha ERD motor response to set a specific patient‐based frequency results more effective in motor rehabilitation procedures (Daly et al., 2009; Marquez‐Chin et al., 2016). Hence, enhancing our knowledge about the spatio‐temporal features underlying the MVF phenomenon may facilitate the EEG‐based BCI application and promote motor rehabilitation for those patients who cannot rely on the correct movement of one limb.

## 5 CONCLUSIONS

In the present work, we tested the hypothesis that MVF illusion may be underpinned by central alpha ERD during movement preparation and execution. Unilateral right finger movements reflected on a mirror produce bilateral central alpha ERD. The results suggest that auditory‐triggered movement preparation and execution during MVF illusion may involve the same somatomotor mechanisms of an actual movement. This may be of interest to develop individualized therapies using MVF illusion for the treatment of chronic pain syndromes associated with motor dysfunction or paralysis.

## AUTHOR CONTRIBUTIONS

**Marco Rizzo:** Data curation; formal analysis; methodology; software; writing – original draft. **Laura Petrini:** Investigation; supervision; writing – review and editing. **Claudio Delpercio:** Formal analysis; software. **Susanna Lopez:** Formal analysis; software. **Lars Arendt‐Nielsen:** Project administration; supervision. **Claudio Babiloni:** Conceptualization; methodology; writing – review and editing.

## Supporting information


**FIGURE S1** EMG activity pattern (mean ± SE) for nine subjects for each condition (Unilateral *M*−, Bilateral *M*−, and Unilateral *M*+). The *Y*‐axes show the power activity in microvolt, whereas the *X*‐axes report the time in seconds. The 0 (zero) represents the auditory cue onset. Of interest, the Bonferroni‐corrected post hoc test showed a statistically significant difference between the right and left hand in the Unilateral *M*+ condition (*p* = 0.000). The right bottom table shows the means (± SE) of the EMG magnitude in microvolt of both hands for each condition.
**FIGURE S2** The figure illustrates a 3 × 2 ANOVA for the occipital electrodes during movement execution (main factors: condition and channel). Results found a significant effect for the factor Condition, indicating a stronger alpha ERD in the Uni *M*+ condition as compared to Uni *M*− and Bil *M*− conditions (*p* < 0.05).
