# Supplementary material for: Mirror visual feedback during unilateral finger movements is related to the desynchronization of cortical electroencephalographic somatomotor alpha rhythms
Source: Psychophysiology. 2022 Jun 3;59(12):e14116. doi: 10.1111/psyp.14116 (PMC9788070; doi:10.1111/psyp.14116)
Supplement: Supplementary file 1 — FIGURE S1 EMG activity pattern (mean ± SE) for nine subjects for each condition (Unilateral M−, Bilateral M−, and Unilateral M+). The Y‐axes show the power activity in microvolt, whereas the X‐axes report the time in seconds. The 0 (zero) represents the auditory cue onset. Of interest, the Bonferroni‐corrected post hoc test showed a statistically significant difference between the right and left hand in the Unilateral M+ condition (p = 0.000). The right bottom table shows the means (± SE) of the EMG magnitude in microvolt of both hands for each condition. FIGURE S2 The figure illustrates a 3 × 2 ANOVA for the occipital electrodes during movement execution (main factors: condition and channel). Results found a significant effect for the factor Condition, indicating a stronger alpha ERD in the Uni M+ condition as compared to Uni M− and Bil M− conditions (p < 0.05). [file PSYP-59-e14116-s001.docx]

**Supplementary materials**

*EMG analysis*

*Methods*

For the EMG recordings, two recording electrodes (Neuroline 720, Ambu, Denmark) were placed on the skin over the first dorsal interosseous (FDI) muscle in both hands. The reference electrode (Neuroline 720, Ambu, Denmark) was placed on the ulnar styloid process (Botelho, Curran, & Lowery, 2019). The EMG activity was acquired using a g.HIamp amplifier (g.tec medical engineering GmbH, Austria) with a sampling frequency of 1,200Hz and notch-filtered to reduce line noise and store data for offline analyses.

Afterwards, the recorded EMG data were rectified by calculating the absolute value. The envelope of the rectified data was obtained by refiltering (2^nd^ order low-pass filter with a cut-off frequency of 1.5 Hz). This procedure produced 80 EMG epochs lasting 4 s each. They were averaged to obtain a measure of EMG activity in the recording conditions. Of note, the EMG recordings were introduced at a later stage of the scientific program and only data from the last nine participants were available.

*Statistical analysis*

A 2-way ANOVA for repeated measures was performed for the EMG data, considering *Condition* (*Unilateral M-, Bilateral M-,* and *Unilateral M+*) and *Hand* (*Left* and *Right*) as factors (p < 0.05). The working hypothesis predicted a 2-way effect of the ANOVA and post hoc tests showing a significant difference in the EMG measures in the left finger between *Bilateral M-* and *Unilateral M+* and *Unilateral M-* (p < 0.05, corrected) and no differences in the left finger between *Bilateral M-* and *Unilateral M+* (p > 0.05, corrected).

*Results*

The ANOVA showed a significant main effect for the factor *Condition* (F(2,16) = 8,640; p = 0.003), indicating greater EMG values during the *Bil M-* condition than *Uni M-* and *Uni M+* conditions. Moreover, there were no significant effects for the factor *Hand* (F(1,18) = 5,051; p > 0.05). The interaction between the factors was significant (F(2,16) = 10,747; p = 0.001). Bonferroni-corrected post hoc (t-paired) tests showed that the EMG magnitude in the *Bil M-* condition is significantly stronger than that in the *Uni M-* condition (p = 0.002) and the *Uni M+* condition (p = 0.003). On the other hand, the EMG magnitude did not differ between the *Uni M+* and *Uni M-* conditions (p = 0.750). Figure S1 shows the mean EMG activity collected from the left and right hands for each condition. Notably, during the unilateral right finger movements performed in the *Uni M+* (experimental) condition, the mean EMG activity was prominent from the moving right hand, while it was negligible from the left still hand. Paired-samples t-test showed that the mean EMG activity recorded from the right and left hands differed significantly (p = 0.000). Of interest, post hoc tests did not find any difference amongst the conditions in the right finger EMG power, demonstrating as the EMG activity was constant across the conditions.

**Figure S1**
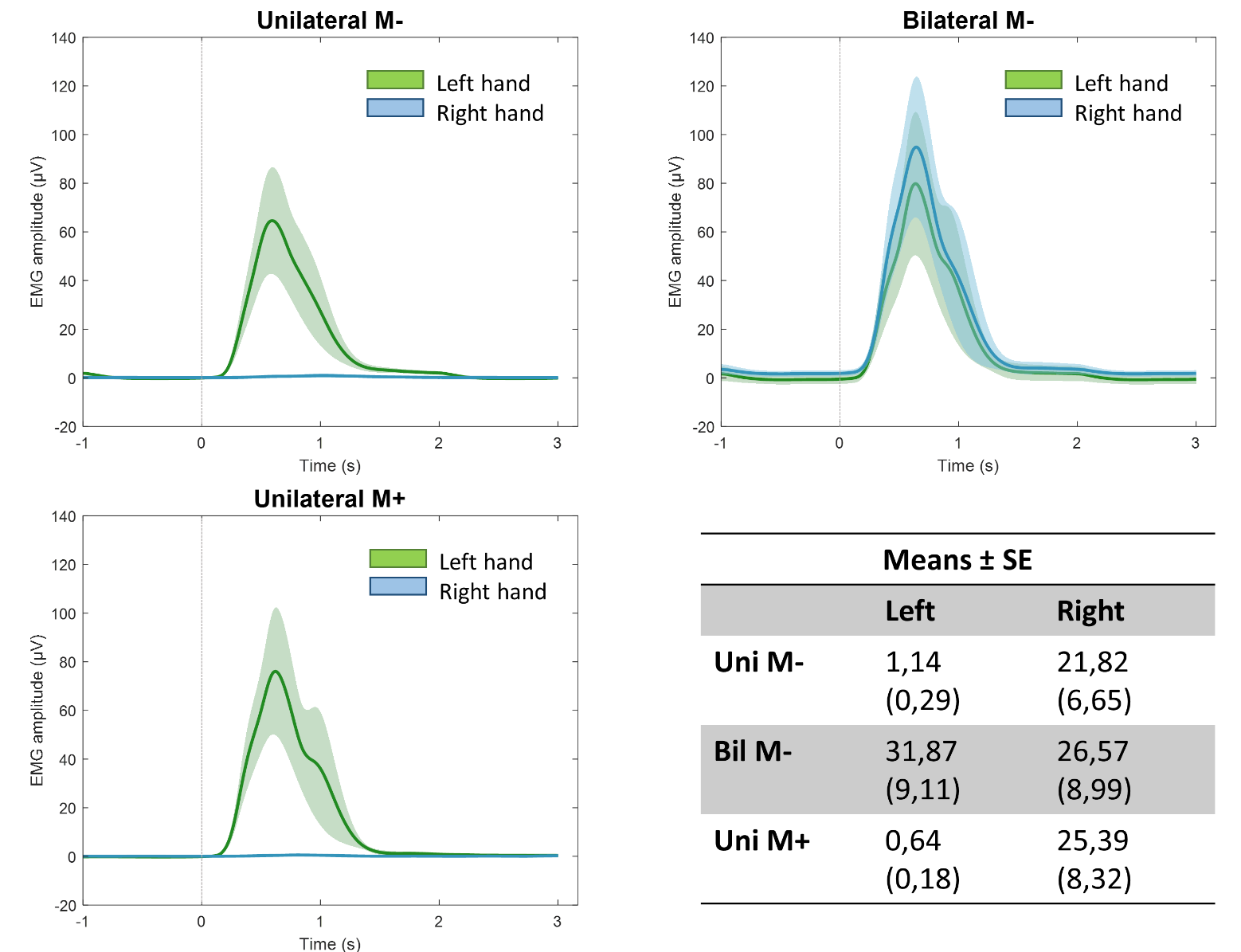


**Figure S1. –** EMG activity pattern (mean ± SE) for nine subjects for each condition (Unilateral M-, Bilateral M-, and Unilateral M+). The Y-axes show the power activity in microvolt, whereas the X-axes report the time in seconds. The 0 (zero) represents the auditory cue onset. Of interest, the Bonferroni-corrected post hoc test showed a statistically significant difference between the right and left hand in the Unilateral M+ condition (p = 0.000). The right bottom table shows the means (± SE) of the EMG magnitude in microvolt of both hands for each condition.

*Occipital analysis*

A two-ways repeated measures ANOVA (3x2) has been performed for the occipital electrodes considering *Condition* (Uni M-, Bil M, and Uni M+) and *Channel* (O1 and O2) as main factors. In the execution phase, the analysis showed a significant effect for *Condition* (F(2,34) = 4,227; p = 0.023; η^2^ = 0,199) and Channel (F(1,17) = 7,408; p = 0.014; η^2^ = 0,304). No significant effects have been shown by the interaction (F(2,34) = 2,135; p = 0.134; η^2^ = 0,112). Data showed a stronger alpha ERD in both the occipital electrodes in the Uni M+ (experimental) condition as compared to the Uni M- and Bil M- (control) conditions.

**Figure S2**


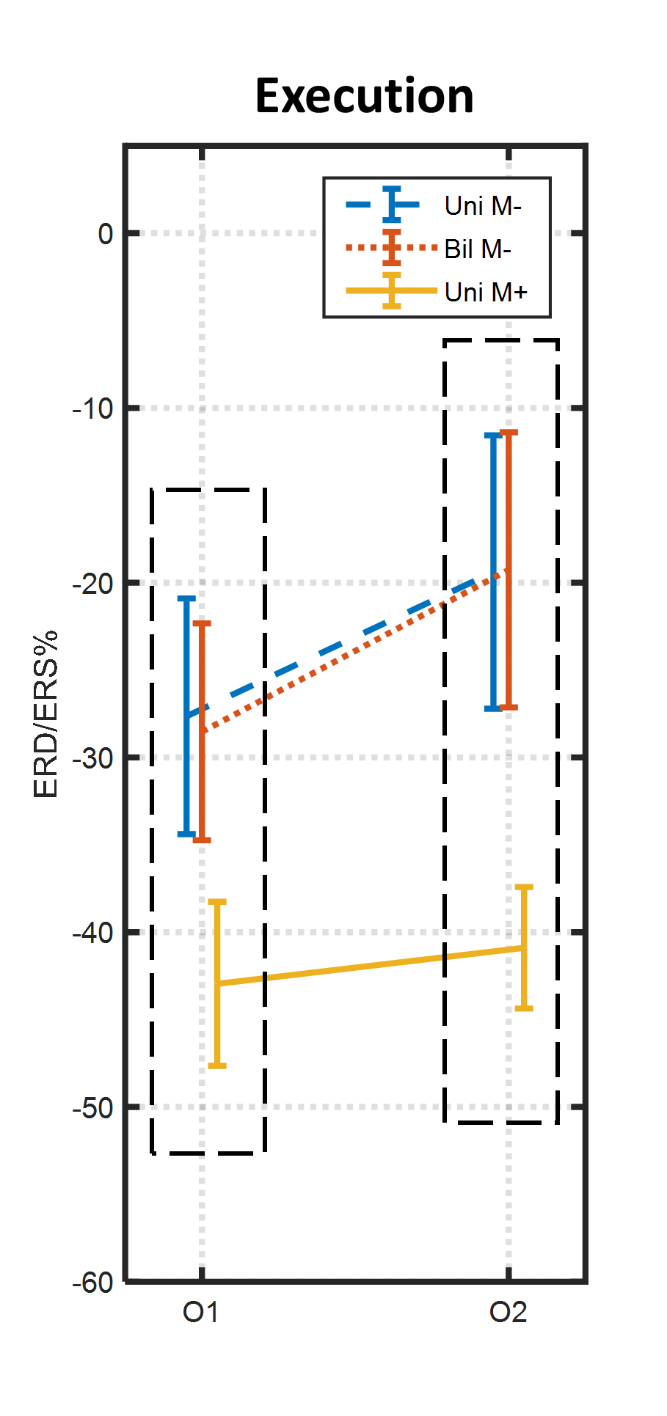


The figure illustrates a 3x2 ANOVA for the occipital electrodes during movement execution (main factors: condition and channel). Results found a significant effect for the factor Condition, indicating a stronger alpha ERD in the Uni M+ condition as compared to Uni M- and Bil M- conditions (p < 0.05).

*References*

Botelho, D. P., Curran, K., & Lowery, M. M. (2019). Anatomically accurate model of EMG during index finger flexion and abduction derived from diffusion tensor imaging. *PLoS Computational Biology*, *15*(8), 1–24. https://doi.org/10.1371/journal.pcbi.1007267
